# Association between Change in the peripheral biomarkers of inflammation, astrocyte activation, and neuroprotection at one week of critical illness and hospital mortality in patients with delirium: A prospective cohort study

**DOI:** 10.1371/journal.pone.0290298

**Published:** 2023-09-01

**Authors:** Sikandar H. Khan, Anthony J. Perkins, Ahmed M. Eltarras, Rosalyn Chi, Ammar A. Athar, Sophia Wang, Noll L. Campbell, Sujuan Gao, Malaz A. Boustani, Babar A. Khan

**Affiliations:** 1 Department of Medicine, Division of Pulmonary, Critical Care, Sleep and Occupational Medicine, Indiana University School of Medicine, Indianapolis, IN, United States of America; 2 Regenstrief Institute, IU Center of Aging Research, Indianapolis, IN, United States of America; 3 Department of Biostatistics and Health Data Science, Indiana University School of Medicine, Indianapolis, IN, United States of America; 4 Department of Internal Medicine, Indiana University School of Medicine, Indianapolis, IN, United States of America; 5 Department of Psychiatry, Indiana University School of Medicine, Indianapolis, IN, United States of America; 6 Department of Pharmacy, Purdue University, Lafayette, IN, United States of America; Bach Mai Hospital, VIET NAM

## Abstract

**Objective:**

In critically ill adults with delirium, biomarkers of systemic inflammation, astrocyte activation, neuroprotection, and systemic inflammation measured at one week of critical illness may be associated with mortality.

**Design:**

Prospective observational study.

**Setting:**

Intensive care unit (ICU).

**Patients:**

178 ICU patients with delirium, alive and remaining in ICU at one week.

**Interventions:**

None

**Measurements and main results:**

Blood samples collected for a pair of previously published, negative, clinical trials were utilized. Samples were collected at study enrollment/ICU admission (Day 1 sample) and one week later (Day 8 sample), and analyzed for interleukins (IL)-6, 8, 10, Insulin-like Growth Factor (IGF), S100 Binding Protein (S100B), Tumor Necrosis Factor Alpha (TNF-A) and C-Reactive Protein (CRP). Delirium, delirium severity, and coma were assessed twice daily using Confusion Assessment Method for the Intensive Care Unit (CAM-ICU), CAM-ICU-7, and Richmond Agitation-Sedation Scale (RASS), respectively. Mortality was assessed until discharge using the electronic medical record. Logistic regression models adjusting for age, sex, severity of illness, comorbidities, sepsis, and randomization status, were used to assess the relationship among biomarkers and mortality. Higher IL-10 quartiles at day 8 were associated with increased odds of hospital mortality (IL-10: OR 2.00 95%CI: 1.1–3.65, p = 0.023). There was a significant interaction between day 1 and day 8 biomarker quartiles only for IL-6. Patients with IL-6 values in the first three quartiles on admission to the ICU that transitioned to higher IL-6 quartiles at day 8 had increased probability of hospital mortality.

**Conclusion:**

In this hypothesis-generating study, higher IL-6 and IL-10 quartiles at one week, and increase in IL-6 from day 1 to day 8 were associated with increased hospital mortality. Studies with larger sample sizes are needed to confirm the mechanisms for these observations.

## Introduction

Delirium is a form of acute brain failure characterized by fluctuations in the level of consciousness, inattention, and/or disorganized thinking [1]. It is highly prevalent in critically ill patients, occurring in nearly 80% of mechanically ventilated older adults [2–4] and adds up to $152 billion in added healthcare costs in the United States each year [5]. Delirium is associated with worse health outcomes including longer duration of mechanical ventilation, longer lengths of hospital stay, lower odds of discharge home, greater than 3-fold risk of death at 6 months, and new cognitive impairment after critical illness [6–8]. Despite this tremendous public health burden, there are significant knowledge gaps in our understanding of intensive care unit (ICU) delirium pathobiology. This scientific gap contributes to the absence of effective pharmacological interventions for delirium [9] and makes management of the delirious patient all the more challenging. For these reasons, there is an urgent scientific need to explore biological mechanisms associated with delirium and mortality [10].

One area of growing scientific interest is the relationship among blood biomarkers associated with delirium duration and severity, and mortality. These biomarkers may reflect major physiological and inflammatory derangements posited to occur in delirium. We previously reported the association among serum biomarkers of systemic inflammation (interleukins (IL) 1, -6, -8, -10, tumor necrosis factor [TNF]–α, C-Reactive Protein), neuroprotection (IGF-1), and astrocyte and glial activation (S-100β) measured at ICU admission and delirium duration, severity, and in-hospital mortality(3). In that study, we found the highest IL-8 and S100B quartiles associated with greater odds of mortality early in the ICU stay. Similarly, prior studies [11] have found CRP measured at admission to be associated with mortality, with median lengths of ICU stay of 6 days before death. Less is known about how these biomarker values change, and their association with mortality, among patients with delirium who remain in the ICU past the first week.

Therefore, we conducted this study to understand the temporal relationship among the above-mentioned biomarkers (collected at two time points: around ICU admission and one week later) and hospital mortality. Our study hypothesis is that higher levels of inflammatory biomarkers at one week would be associated with increased mortality by hospital discharge.

## Materials and methods

This is a prospective observational study utilizing blood samples collected for two connected clinical trials (detailed below). The study received ethical approval from the Institutional Review Board at Indiana University (IUIRB). All participants provided written informed consent (IUIRB Protocol Approval # 1010002428, Pharmacological Management of Delirium (PMD), Full Board Study, Initial Approval February 12, 2009). The study was conducted in accordance with ethical standards of the local IRB and Helsinki Declaration of 1975. The parent study (PMD) clinical trial registration number is NCT00842608.

### Study setting and population

The participants in this study consisted of patients who were admitted to the medical-surgical intensive care units of three tertiary downtown Indianapolis, Indiana, hospitals affiliated with the Indiana University School of Medicine, and who were enrolled in either the Pharmacological Management of Delirium (PMD) trial [8] or the Deprescribing in the Pharmacologic Management of Delirium (De-PMD) trial [12] from March 2009 to January 2015. The PMD and De-PMD studies were a pair of previously published, negative, NIH-funded randomized clinical trials testing the efficacy of low-dose haloperidol combined with deprescribing of anticholinergics and benzodiazepines in reducing delirium duration and severity.

Full eligibility criteria for the trials have been previously published [8,12]. Briefly, patients were enrolled if they were: 1) admitted to the ICU for greater than or equal to 24 hours, 2) were ≥ 18 years of age, 3) screened positive for delirium based on the Richmond Agitation-Sedation Scale (RASS)(13) and the Confusion Assessment Method for the ICU (CAM-ICU)(14), and 4) English-speaking. Patients meeting any of the following criteria were excluded: 1) history of severe mental illness, 2) severe cognitive impairment or severe dementia per electronic medical records, 3) alcohol related delirium, 4) aphasic stroke, 5) pregnant or nursing, or 6) previously enrolled in the study during a prior ICU hospitalization.

Patients were included in the current study if they were enrolled in the PMD/de-PMD trials and had blood samples collected at two time points: 1) a baseline sample: “Day 1”, defined as having been collected within 24 hours of study enrollment (enrollment occurred soon after admission to the ICU); and, 2) a follow-up sample: “Day 8” obtained seven days after enrollment if the patient was alive and still admitted to the ICU. Therefore, inclusion in the present study required availability of two blood samples drawn one week apart from patients admitted to ICU.

#### Biomarkers

As described in prior publications[3], the following biomarkers were selected *a priori* based on an extensive review of the literature and the neuroinflammatory hypothesis of ICU delirium [3,13,14]: A) systemic inflammation: interleukin (IL)-1, -6, -8, -10, tumor necrosis factor-α (TNF-α), and C-Reactive Protein (CRP); B) neuroproliferation: insulin-like growth factor-1 (IGF-1); and, C) astrocyte and glial cell activation: S-100β.

#### Delirium/Coma-free days

Consistent with other high impact publications and to mitigate confounding by death [6,15], we measured delirium duration as Delirium/coma-free days (DCFD) from ICU admission until hospital discharge. DCFD are the number of days that a patient was alive and free of delirium or coma. Patients who died before day 30 had their subsequent delirium-/coma-free days counted as 0. Patients who were discharged alive before day 8 or 30 had the remaining days counted as delirium-/coma-free. Delirium and coma were assessed twice daily (until hospital discharge or death) using the RASS and CAM-ICU [16,17].

#### Delirium severity

We assessed delirium severity twice daily (until death or discharge) using the CAM-ICU-7 [18] from ICU admission until hospital discharge. The CAM-ICU-7 is a 7-point scale that is derived from the CAM-ICU and RASS. On this scale, 0–2 represents no delirium, 3–5 is mild to moderate delirium, and 6–7 corresponds to severe delirium. For delirium severity by day 8 we computed average of the mean CAM-ICU-7 from day 1 to day 8, and for delirium severity by discharge we computed average of the mean CAM-ICU-7 from day 1 until day of discharge.

### Outcome measures

#### Mortality

The primary outcome was mortality by hospital discharge. Data for mortality were retrieved from the electronic medical record (EMR).

#### Other data collection

Additional variables obtained from the EMR are shown in S1 Table.

#### Statistical analysis

Biomarkers with values below the detectable limit were imputed with the midpoint between 0 and the minimum detectable limit. We used Spearman correlation coefficients to assess the relationship between biomarkers, delirium severity and delirium duration. We used Wilcoxon Rank-Sum tests to determine if biomarker values differed by mortality status. To minimize the influence of potential outliers in our models, we categorized each biomarker into quartile groups based on day 1 values of the 321 patients with baseline data. We used logistic regression to assess the relationship between biomarker quartiles, change in biomarker quartiles and in hospital mortality while adjusting for age, sex, APACHE-II, Charlson Comorbidity Index, sepsis, and intervention status. We also examined interaction terms between day 1 and day 8 quartiles for each of the biomarkers. Wilcoxon Rank-Sum tests and Fisher’s Exact tests were used to test for difference between patients with and without biomarker values at one week. All analyses were conducted using SAS v9.4 software (SAS Institute, Cary, NC).

## Results and discussion

A total of 178 patients had blood samples from both baseline (ICU admission) and one week after and were included in the analysis. **Table 1** shows detailed patient demographics, median biomarker values, delirium outcomes and lengths of stay of our study cohort. All patients in the cohort had delirium, with a median delirium duration of 3 days (IQR 1–5). Death occurred in 10.7% (n = 19) during index hospitalization (see Table 1).

**Table 1:** Patient characteristics and clinical outcomes of the study sample.

Variable	Study Cohort* (n = 178)
**Age median (IQR)**	61.0 (53.5, 70.1)
**Female n (%)**	100 (56.2)
**African American n (%)**	86 (48.3)
**Hispanic n (%)**	2 (1.1)
**Education (years)**	12.0 (10.0, 12.0)
APACHE-II* median (IQR)	21.0 (16.0, 27.0)
**Charlson Comorbidity Index median (IQR)**	3.0 (1.0, 5.0)
**Activities of Daily Living (ADL) median (IQR)**	6.0 (5.0, 6.0)
**Instrumental Activities of Daily Living (IADL) median (IQR)**	7.0 (4.0, 8.0)
**IQCODE median (IQR)**	3.0 (3.0, 3.3)
**Mechanically Ventilated n (%)**	135 (75.8)
**ICU Location n (%)**	
**Medical ICU**	122 (68.5)
**Surgical ICU**	47 (26.4)
**Intermediate ICU**	9 (5.1)
**Primary Admission Diagnoses n (%)**	
**Acute Respiratory Failure and/or Sepsis**	96 (53.9)
**Neurological/encephalopathy**	17 (9.6)
**Other**	65 (36.5)
**Biomarker values at Day 1; median (IQR)**	
**IL-1 pg/ml**	10.0 (4.2, 36.1)
IL-6 pg/ml	28.0 (11.4, 59.4)
**IL-8 pg/ml**	32.0 (18.2, 61.5)
**IL-10 pg/ml**	11.7 (3.9, 27.5)
TNF-α pg/ml	12.0 (7.4, 19.0)
**S-100β ng/ml**	0.1 (0.1, 0.2)
IGF-1 ng/ml	37.0 (26.3, 65.1)
**C-Reactive Protein μg/ml**	29.2 (21.1, 46.7)
**Delirium Duration and Severity median (IQR)**	
**Delirium/coma free days by 30 days**	24.0 (17.0, 28.0)
**Delirium severity by discharge**	2.9 (1.6, 4.5)
**Length of Stay, Discharge Disposition, and Mortality**	
**ICU length of stay in days**	13.0 (10.0, 22.0)
**Hospital length of stay in days**	16.0 (12.0, 28.0)
**Discharged home n (%)**	49 (27.5)
**In-hospital mortality n (%)**	19 (10.7)

*All patients in the cohort had two blood samples collected as follows: Baseline sample was collected at study enrollment soon after ICU admission. Follow-up blood sample was collected if patient was alive and remained in ICU one week after baseline sample. Delirium severity was assessed twice daily by Confusion Assessment Method for the ICU-7. ADL: Activities of Daily Living; APACHE: Acute Physiology and Chronic Health Evaluation Score; CRP: C-Reactive Protein; IADL: Instrumental Activities of Daily Living. IL: Interleukin. IGF: Insulin like growth factor-1. IQCODE: Informant Questionnaire on Cognitive Decline Elderly. TNF-A: Tumor Necrosis Factor Alpha.

As shown in **S1 Table**, there were important clinical, biomarker, and outcomes differences between the current study sample (patients who remained in the ICU at one week) and patients excluded due to having only blood samples collected at Day 1 (i.e., these patients were no longer in the ICU at day 8 due to death or discharge). Patients who remained in the ICU at one week (the study cohort) had significantly higher rates of mechanical ventilation, higher median values IL-1, IL-6, IL-8, IL-10, TNF-A, S100B, longer delirium duration and severity, and greater lengths of ICU and hospital stay (see S1 Table).

### Association of biomarker levels at day 8 and mortality at discharge (Table 2)

Among delirious, critically ill patients remaining in the ICU for at least 7 days, median levels of IL-6 (deceased: 44.3, IQR 19.1–71.6 vs. alive: 12.4, IQR 5.0–30.6), IL-8 (deceased: 58.7, IQR 29.1–97.3 vs. alive: 30.0, IQR 16.6–45.0), and IL-10 (deceased: 15.3, IQR 9.9–45.7 vs. alive: 6.8, IQR 1.1–22.4) measured on day 8 were significantly higher in patients who died during the hospitalization compared to those who survived. Unadjusted results of univariate comparisons of day 8 biomarkers by mortality are shown in **Table 2**.

**Table 2:** Univariate comparison of day 8 biomarker values by mortality status at discharge.

Comparison of Day 8 Biomarkers by Mortality Status (Alive vs Dead)			
	**Day 8**		
	**Alive** **(n = 159)**	**Dead** **(n = 19)**	**P-value**
Biomarkers of Inflammation			
IL-1	12.0 (4.2, 39.0)	16.7 (4.2, 54.4)	0.696
IL-6	12.4 (5.0, 30.6)	44.3 (19.1, 71.6)	0.001
IL-8	30.0 (16.6, 45.0)	58.7 (29.1, 97.3)	0.012
IL-10	6.8 (1.1, 22.4)	15.3 (9.9, 45.7)	0.001
TNF-α	12.6 (8.1, 19.3)	16.1 (7.3, 20.3)	0.504
CRP	25.6 (15.9, 1.2)	24.4 (20.5, 53.6)	0.445
Biomarkers of Astrocyte and Glial Cell Activation			
S-100β	0.1 (0.1, 0.2)	0.2 (0.1, 0.6)	0.009
Biomarkers of Neuroprotection			
IGF-1	41.4 (25.0, 67.5)	27.5 (21.9, 39.2)	0.009

Median and IQR values of each biomarker shown. CRP: C-Reactive Protein. IGF: Insulin like growth factor-1. IL: Interleukin. TNF-A: Tumor Necrosis Factor Alpha.

We also found higher median values of S100β at day 8 (deceased: 0.2, IQR 0.1–0.6 vs. alive: 0.1, IQR 0.1–0.2) in patients who died compared to those who survived. Conversely, patients who died had lower median levels of IGF-1 at day 8 (deceased: 27.5, IQR 21.9–39.2 vs. alive: 41.4, IQR 25.0–67.5) compared to survivors, as shown in **Table 2**.

### Association of biomarkers with hospital mortality (Figs 1 and 2)

**Fig 1:**
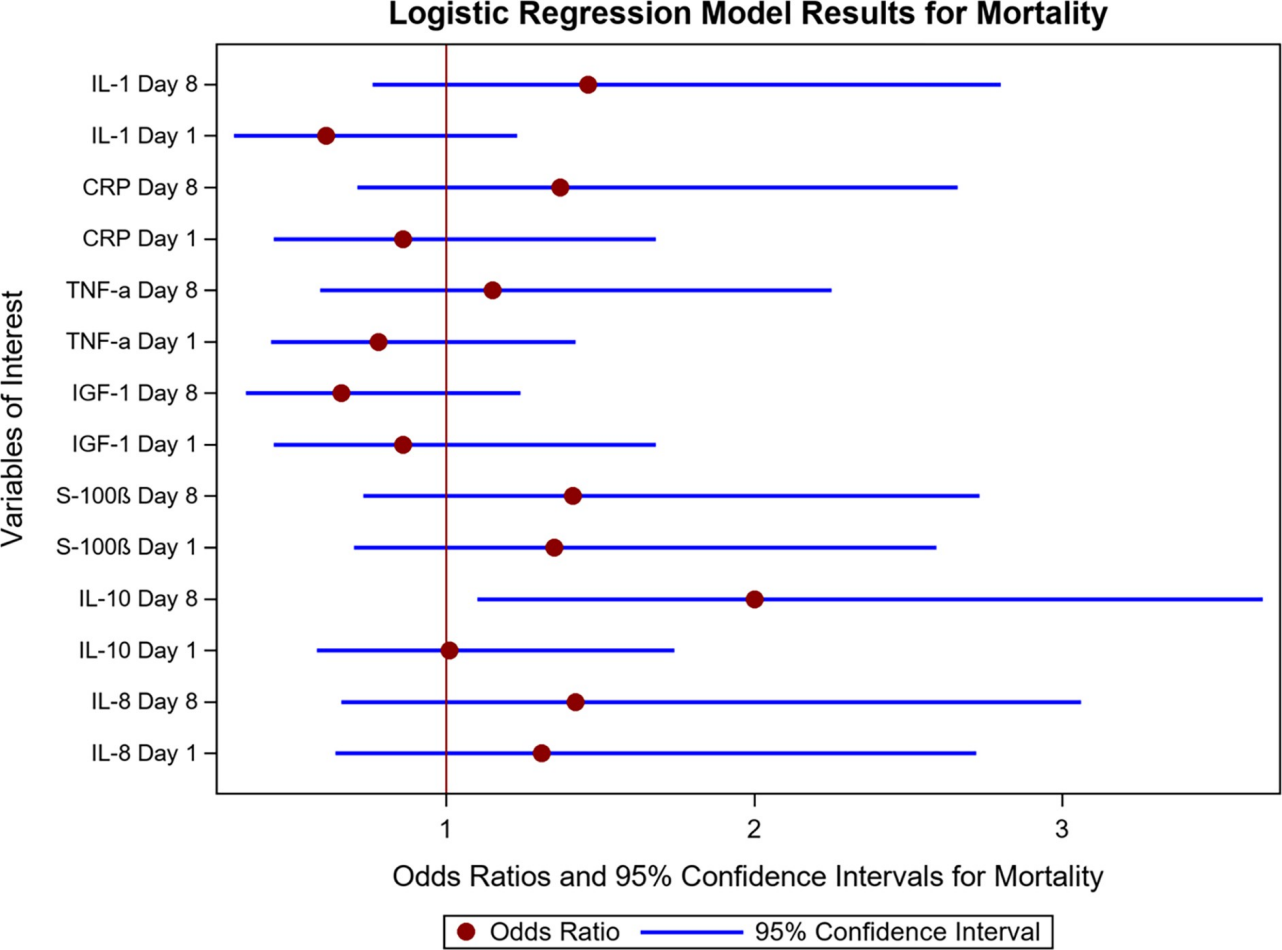
Relationship among biomarkers at day 1 and day 8 and odds of hospital mortality. As shown in Fig 1, higher IL-10 quartiles at day 8 were associated with increased odds of hospital mortality (IL-10: OR 2.00 95%CI: 1.1–3.65, p = 0.023). The results did not change when we added day 8 mechanical ventilation status to the regression model, and higher IL-10 quartiles at day 8 remained associated with increased mortality (OR 1.92 95%CI 1.05–3.51, p = 0.033). As mentioned above, there were no significant interactions between day 1 and day 8 biomarker quartiles for IL-10.

**Fig 2:**
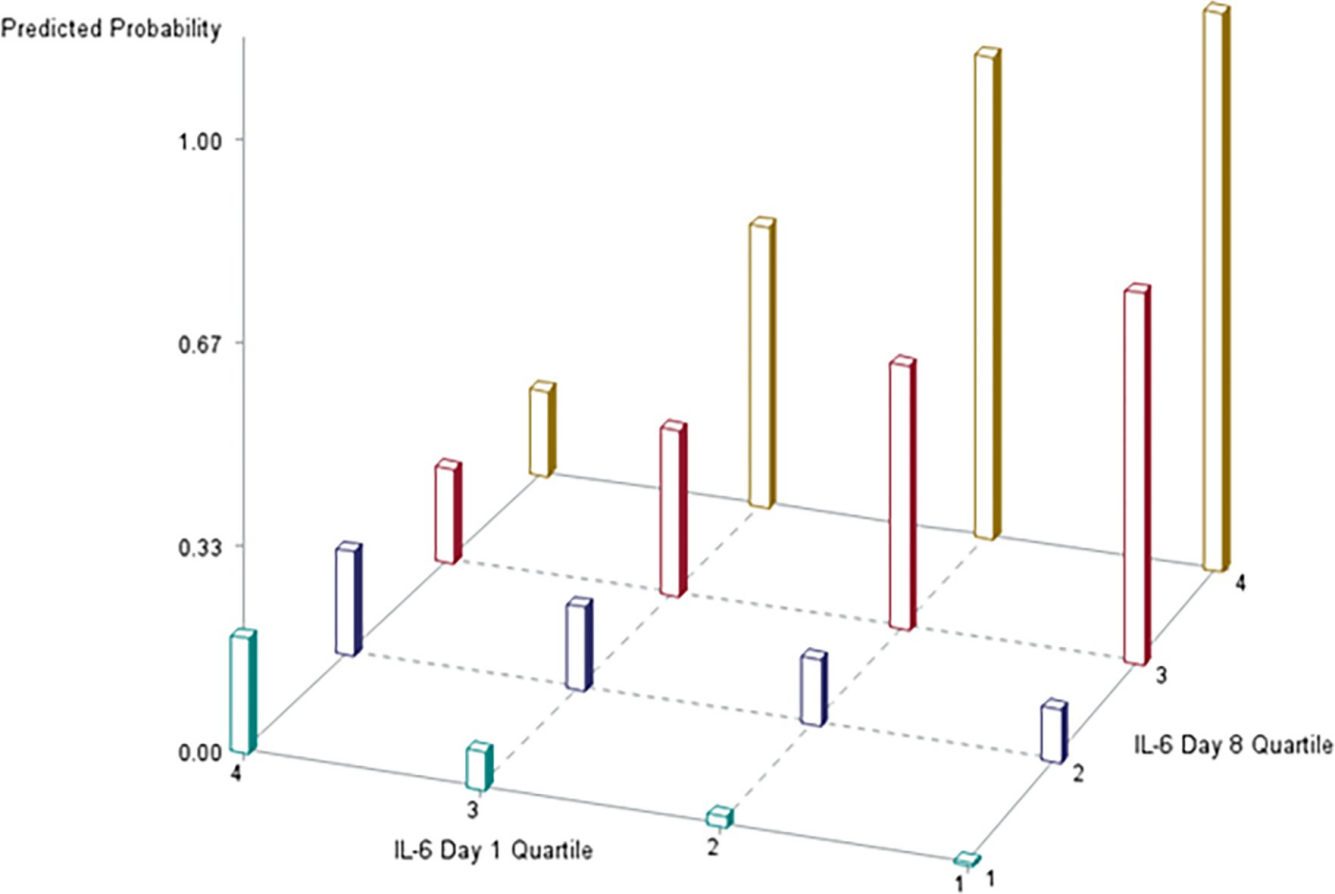
Relationship among interleukin-6 quartiles at day 1 and day 8 and predicted probability* of hospital mortality. *We computed predicted probability of mortality assuming a female usual care patient with sepsis, an APACHE II score of 20, age 60, and a Charlson Comorbidity Index of 3.

We performed a logistic regression model to assess the relationship between biomarker quartiles at the two time points and mortality while adjusting for age, sex, APACHE-II, Charlson Comorbidity Index, sepsis, and intervention status (randomization status in the parent trial). Results of our model with day 1 and day 8 biomarkers are shown in S2 Table. There was only a significant interaction between day 1 and day 8 biomarker quartiles for IL-6. Results for the biomarkers are shown in **Figs 1 and 2**.

There was a significant interaction between day 1 and day 8 biomarker quartiles for IL-6, and remained significant when day 8 mechanical ventilation status was added to the model. As illustrated in Fig 2, patients with IL-6 values in the first three quartiles on admission to the ICU that transitioned to higher IL-6 quartiles at day 8 were at increased probability of hospital mortality. The probability of mortality was highest in patients who transitioned from quartile 1 to 4, and 2 to 4. However, for patients with the highest IL-6 quartile at day 1 who survived until day 8, predicted mortality was similar across day 8 quartiles.

### Correlation of biomarkers at day 1 and day 8 with delirium duration and severity (S3 Table)

We found higher day 1 values of IL-6 weakly correlated with greater delirium severity by day 8, and discharge, and weakly correlated with greater delirium/coma duration both by day 8 and by day 30. Higher IL-10 values on day 1were weakly correlated with greater delirium/coma duration by day 30. When measured on day 8, higher IL-10 values were weakly correlated with greater delirium/coma duration by 30 days. Spearman correlations for all the biomarkers are shown in **S3 Table**.

## Discussion

Delirium and coma are associated with increased risk of death for up to 1 year after acute illness [19], but differences in the pathobiology underlying this observation is not fully defined. As a result, there is a strong need to identify endotypes associated with ICU delirium and mortality. In this hypothesis-generating study, we sought to measure the relationship among biomarkers of systemic inflammation, astrocyte activation, and neuroprotection, and hospital mortality in a cohort of delirious patients remaining in the ICU past 7 days. Our study found that higher biomarker quartiles of IL-10 on day 8 were associated with increased mortality. We also found a significant interaction between IL-6 quartiles on day 1 and day 8, and that for patients with low IL-6 values at day 1, a transition to the highest IL-6 quartiles by day 8 was associated with increased hospital mortality. We did not see this relationship among patients who were in the highest IL-6 quartile on day 1, likely as our study methodology required survival until day 8 (and we discuss this further in our limitations). Studies with larger sample sizes are needed to confirm the mechanisms for these observations.

Our findings add nuance to prior biomarker work showing greater quartiles of CRP, IL-8, and S-100β measured at delirium onset were associated with increased in-hospital mortality [3]. Our current analysis exclusively focused on patients who remained alive and in the ICU past one week, thereby allowing us to explore change in biomarker levels over time. Our results suggest possible utility in measuring IL-6 and IL-10 at two time points for delirious patients who remain in the ICU past the first week of admission, but larger follow up studies incorporating daily severity of illness risk measures are needed to determine whether these biomarkers identify patients who will die vs. those who may survive.

Numerous studies examining the association of biomarkers and mortality in adult and pediatric patients with sepsis and burn injuries have recently been published [20–23]. We chose to study a panel of biomarkers given their biological plausibility in delirium pathophysiology; these biomarkers may be associated with systemic inflammation, neuronal injury and loss of neuronal protection, and astrocyte and microglial activation within the central nervous system [3,24]. Our methodological approach utilized blood samples from two time points [6,25–27] to better account for the dynamic nature of an ICU patient’s clinical trajectory, response to treatment, or the development of unexpected complications. The repeated measurement of biomarkers allowed us to explore the change in biomarker values and their relationship with mortality.

Our study did have some important limitations. First, all patients in this study had delirium, as our cohort was derived from a prior study that was primarily designed to explore biomarkers in relation to delirium severity and duration. Hence, we cannot draw conclusions about whether these biomarker relationships differ by delirium status, and our results may not be generalizable to ICU patients without delirium. Second, our findings represent association rather than causation, and further studies are needed to shed light on the pathophysiology linking specific biomarkers with mortality. Third, despite high severity of illness, the mortality in our study was only 11% which may have underpowered our ability to fully detect the association of biomarkers with mortality. The low mortality may be related to the design of our study which selected patients who survived until at least one week of ICU, and the duration of follow-up in the study. Fourth, it is possible that any associations we found were due to chance, as our study was meant to be hypothesis-generating, and we did not statistically adjust for the number of tests performed. Finally, while our models included variables for severity of illness at day 1, sepsis, comorbidities, and day 8 mechanical ventilation status, they did not include day 8 severity of illness scores. Further studies with a larger cohort of patients, repeated biomarker measurements, severity of illness at multiple time points, and longer follow-up are needed for validation of our results.

## Conclusions

In conclusion, among critically ill patients with delirium, our hypothesis-generating study suggests levels of IL-6 and IL-10 at one week of ICU stay may be associated with mortality. Future research should be focused on elucidating the pathophysiology involving biomarkers and mortality.

## Supporting information

S1 Table Comparison of characteristics and clinical outcomes between patients in the study cohort and those with samples collected only at day 1 (excluded from current study). Data presented as median (IQR) or n (%) unless otherwise specified. CRP: C-Reactive Protein. IGF: Insulin like growth factor-1. IL: Interleukin. TNF-A: Tumor Necrosis Factor Alpha. ADL: Activities of Daily Living. APACHE: Acute Physiology and Chronic Health Evaluation Score. CRP: C-Reactive Protein. IADL: Instrumental Activities of Daily Living. IL: Interleukin. IGF: Insulin like growth factor-1. IQCODE: Informant Questionnaire on Cognitive Decline Elderly. IQR: Interquartile Range. TNF-A: Tumor Necrosis Factor Alpha.
(DOCX)

S2 Table Biomarkers measured at day 1 and day 8 and odds of in-hospital mortality. Logistic Regression Models adjusted for age, gender, APACHE, Charlson, Sepsis, Intervention Status in the parent trial. IL: Interleukin. TNF-A: Tumor Necrosis Factor Alpha. IGF: Insulin like growth factor-1. CRP: C-Reactive Protein.
(TIF)

S3 Table Correlation of biomarker values with assorted outcomes. *P<0.05. Spearman correlations shown. **Mean delirium severity by discharge (computed as average of mean CAM-ICU-7 scores by discharge), ***Duration of delirium or coma beyond day 8 (as measured by number of delirium- and coma-free days at Day 30 minus delirium- and coma-free days at Day 8). CRP: C-Reactive Protein. IGF: Insulin like growth factor-1. IL: Interleukin. TNF-A: Tumor Necrosis Factor Alpha.
(TIF)
