# Supplementary material for: Association between Change in the peripheral biomarkers of inflammation, astrocyte activation, and neuroprotection at one week of critical illness and hospital mortality in patients with delirium: A prospective cohort study
Source: PLoS One. 2023 Sep 1;18(9):e0290298. doi: 10.1371/journal.pone.0290298 (PMC10473496; doi:10.1371/journal.pone.0290298)
Supplement: S1 Table — Data presented as median (IQR) or n (%) unless otherwise specified. CRP: C-Reactive Protein. IGF: Insulin like growth factor-1. IL: Interleukin. TNF-A: Tumor Necrosis Factor Alpha. ADL: Activities of Daily Living. APACHE: Acute Physiology and Chronic Health Evaluation Score. CRP: C-Reactive Protein. IADL: Instrumental Activities of Daily Living. IL: Interleukin. IGF: Insulin like growth factor-1. IQCODE: Informant Questionnaire on Cognitive Decline Elderly. IQR: Interquartile Range. TNF-A: Tumor Necrosis Factor Alpha. (DOCX) [file pone.0290298.s001.docx]

**S1 Table**. **Comparison of Characteristics and Clinical Outcomes Between Patients in the Study Cohort and Those with Samples Collected Only at Day 1 (Excluded from Current Study)**

| **Variable** | **Patients with Only Day 1 Blood Sample – Patients Excluded from current study***  **(n=143)** | **Patients with Day 1 and Day 8 Blood Sample - Study Cohort****  **(n=178)** | **P-value** |
| --- | --- | --- | --- |
| **Age median (IQR)** | 60.4 (49.6, 69.6) | 61.0 (53.5, 70.1) | 0.434 |
| **Female n (%)** | 79 (55.2) | 100 (56.2) | 0.910 |
| **African American n (%)** | 72 (50.3) | 86 (48.3) | 0.737 |
| **Hispanic n (%)** | 0 (0.0) | 2 (1.1) | 0.504 |
| **Education (years)** | 12.0 (10.0, 12.0) | 12.0 (10.0, 12.0) | 0.445 |
| **APACHE-II** | 20.0 (13.0, 26.0) | 21.0 (16.0, 27.0) | 0.157 |
| **Charlson Comorbidity Index** | 2.0 (1.0, 4.0) | 3.0 (1.0, 5.0) | 0.054 |
| **Activities of Daily Living (ADL)** | 6.0 (5.0, 6.0) | 6.0 (5.0, 6.0) | 0.573 |
| **Instrumental Activities of Daily Living (IADL)** | 8.0 (3.0, 8.0) | 7.0 (4.0, 8.0) | 0.237 |
| **IQCODE** | 3.0 (3.0, 3.3) | 3.0 (3.0, 3.3) | 0.610 |
| **Mechanically Ventilated n (%)** | 61 (42.7) | 135 (75.8) | <0.001 |
| **ICU Location** |  |  | 0.055 |
| Medical ICU n (%) | 97 (67.8) | 122 (68.5) |  |
| Surgical ICU n (%) | 29 (20.3) | 47 (26.4) |  |
| Intermediate ICU n (%) | 17 (11.9) | 9 (5.1) |  |
| **Primary Admission Diagnoses** |  |  | 0.297 |
| Acute Respiratory Failure and/or Sepsis n (%) | 71 (49.7) | 96 (53.9) |  |
| Neurological/Encephalopathy n (%) | 9 (6.3) | 17 (9.6) |  |
| Other n (%) | 63 (44.1) | 65 (36.5) |  |

**S1 Table. Comparison of Characteristics and Clinical Outcomes Between Patients in the Study Cohort and Those with Samples Collected Only at Day 1 (Excluded from Current Study)**

| **Biomarker values at Day 1 median (IQR)** | | | |
| --- | --- | --- | --- |
| IL-1 pg/ml | 4.2 (4.2, 25.1) | 10.0 (4.2, 36.1) | 0.014 |
| IL-6 pg/ml | 11.5 (4.5, 33.6) | 28.0 (11.4, 59.4) | <0.001 |
| IL-8 pg/ml | 20.9 (11.7, 55.1) | 32.0 (18.2, 61.5) | 0.001 |
| IL-10 pg/ml | 6.0 (1.3, 17.0) | 11.7 (3.9, 27.5) | 0.001 |
| TNF-α pg/ml | 9.2 (4.8, 15.3) | 12.0 (7.4, 19.0) | 0.001 |
| S-100β ng/ml | 0.1 (0.0, 0.2) | 0.1 (0.1, 0.2) | 0.003 |
| IGF-1 ng/ml | 35.3 (25.6, 60.6) | 37.0 (26.3, 65.1) | 0.526 |
| C-Reactive Protein µg/ml | 32.3 (19.1, 38.0) | 29.2 (21.1, 46.7) | 0.239 |
| **Delirium Outcomes median (IQR)** | | | |
| Delirium duration in days (from admission) | 1.0 (0.0, 2.0) | 3.0 (1.0, 5.0) | <0.001 |
| Delirium/coma free days by one week | 6.0 (2.0, 8.0) | 3.0 (1.0, 6.0) | <0.001 |
| Delirium/coma free days by 30 days | 28.0 (24.0, 30.0) | 24.0 (17.0, 28.0) | <0.001 |
| Delirium severity by one week mean (SD) | 2.8 (1.0, 5.5) | 4.3 (2.5, 5.8) | 0.002 |
| Delirium severity by discharge mean (SD) | 2.1 (0.8, 4.7) | 2.9 (1.6, 4.5) | 0.049 |
| **Length of Stay and Mortality Outcomes** | | | |
| ICU length of stay (days) | 6.0 (4.0, 10.0) | 13.0 (10.0, 22.0) | <0.001 |
| Hospital length of stay (days) | 7.0 (5.0, 12.0) | 16.0 (12.0, 28.0) | <0.001 |
| Discharged home n (%) | 68 (47.6) | 49 (27.5) | <0.001 |
| In-hospital mortality n (%) | 16 (11.2) | 19 (10.7) | 1.000 |

Data presented as median (IQR) or n (%) unless otherwise specified.
CRP: C-Reactive Protein. IGF: Insulin like growth factor-1. IL: Interleukin. TNF-A: Tumor Necrosis Factor Alpha. ADL: Activities of Daily Living. APACHE: Acute Physiology and Chronic Health Evaluation Score. CRP: C-Reactive Protein. IADL: Instrumental Activities of Daily Living. IL: Interleukin. IGF: Insulin like growth factor-1. IQCODE: Informant Questionnaire on Cognitive Decline Elderly. IQR: Interquartile Range. TNF-A: Tumor Necrosis Factor Alpha.
