# Supplementary figures and images for: Association between Change in the peripheral biomarkers of inflammation, astrocyte activation, and neuroprotection at one week of critical illness and hospital mortality in patients with delirium: A prospective cohort study
Source: PLoS One. 2023 Sep 1;18(9):e0290298. doi: 10.1371/journal.pone.0290298 (PMC10473496; doi:10.1371/journal.pone.0290298)

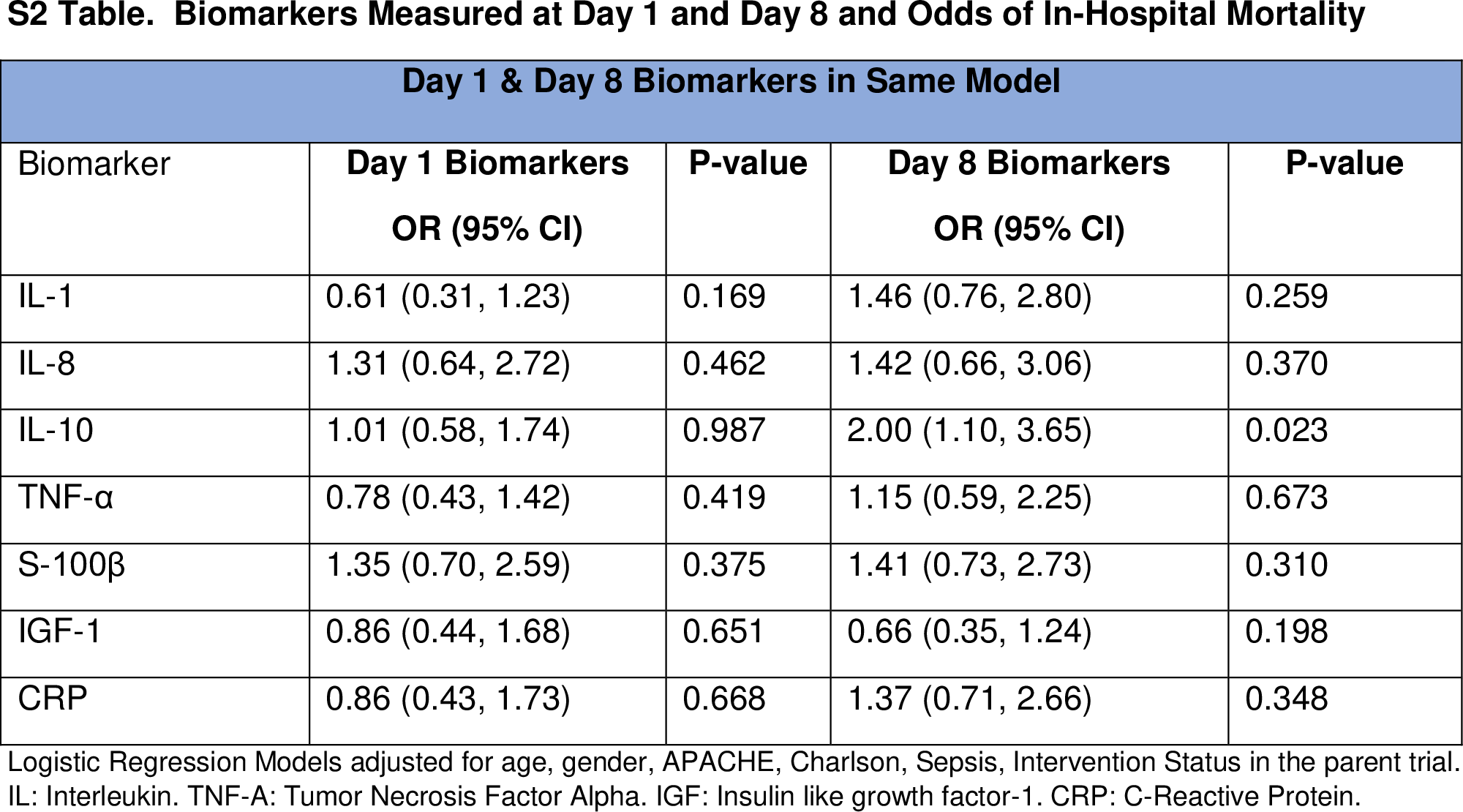

Supplement: S2 Table — Logistic Regression Models adjusted for age, gender, APACHE, Charlson, Sepsis, Intervention Status in the parent trial. IL: Interleukin. TNF-A: Tumor Necrosis Factor Alpha. IGF: Insulin like growth factor-1. CRP: C-Reactive Protein. (TIF) [file pone.0290298.s002.tif]

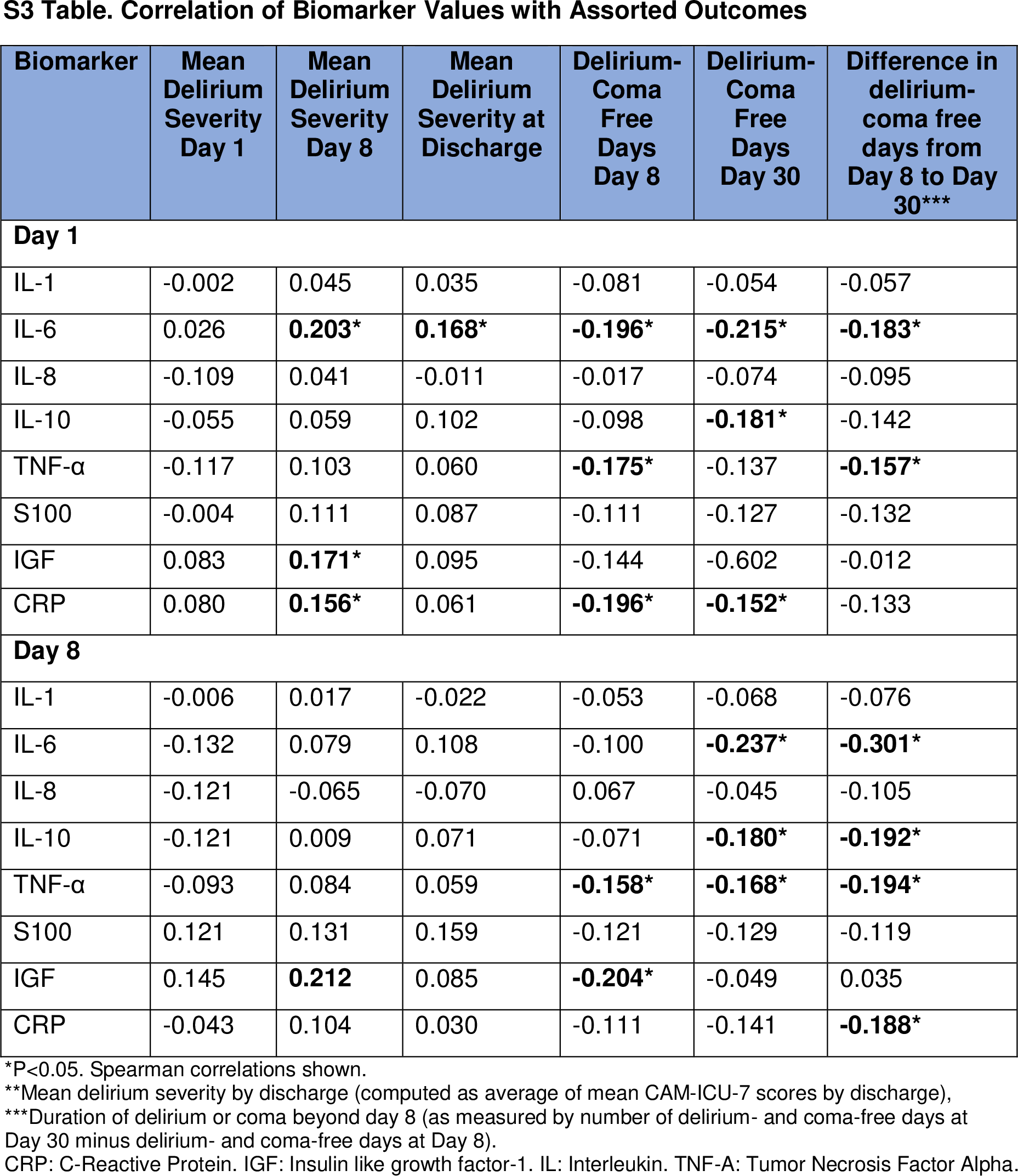

Supplement: S3 Table — *P<0.05. Spearman correlations shown. **Mean delirium severity by discharge (computed as average of mean CAM-ICU-7 scores by discharge), ***Duration of delirium or coma beyond day 8 (as measured by number of delirium- and coma-free days at Day 30 minus delirium- and coma-free days at Day 8). CRP: C-Reactive Protein. IGF: Insulin like growth factor-1. IL: Interleukin. TNF-A: Tumor Necrosis Factor Alpha. (TIF) [file pone.0290298.s003.tif]
